# The needs and opportunities for housing improvement for malaria control in southern Tanzania

**DOI:** 10.1186/s12936-023-04499-1

**Published:** 2023-02-27

**Authors:** Ramadhani M. Bofu, Ellen M. Santos, Betwel J. Msugupakulya, Najat F. Kahamba, Joseph D. Swilla, Rukiyah Njalambaha, Ann H. Kelly, Javier Lezaun, Nicola Christofides, Fredros O. Okumu, Marceline F. Finda

**Affiliations:** 1 Department of Environmental Health and Ecological Sciences, Ifakara Health Institute, P. O. Box 53, Ifakara, Tanzania; 2 School of Life Sciences and Bioengineering, The Nelson Mandela African Institution of Science and Technology, P. O. Box 447, Arusha, Tanzania; 3 Mpwapwa Institute of Health and Allied Sciences, The Ministry of Health, P.O. Box 743, Dodoma, Tanzania; 4 Department of Applied Health, Southern Illinois University Edwardsville, Edwardsville, USA; 5 Department of Vector Biology, Liverpool School of Tropical Medicine, Liverpool, UK; 6 School of Biodiversity, One Health and Veterinary Medicine, University of Glasgow, Glasgow, G128QQ UK; 7 Department of Molecular Biology and Biotechnology, University of Dar es Salaam, Dar es Salaam, Tanzania; 8 Department of Global Health and Social Medicine, King’s College London, London, UK; 9 Institute for Science, Innovation, and Society, School of Anthropology and Museum Ethnography, University of Oxford, Oxford, UK; 10 School of Public Health, Faculty of Health Sciences, University of the Witwatersrand, 1 Smuts Avenue, Braamfontein, Johannesburg, 2000 South Africa

**Keywords:** Housing improvement, Need, Magnitude, Opportunities, Malaria control

## Abstract

**Background:**

Malaria disproportionately affects low-income households in rural communities where poor housing is common. Despite evidence that well-constructed and mosquito-proofed houses can reduce malaria risk, housing improvement is rarely included in malaria control toolboxes. This study assessed the need, magnitude, and opportunities for housing improvement to control malaria in rural Tanzania.

**Methods:**

A mixed-methods study was conducted in 19 villages across four district councils in southern Tanzania. A structured survey was administered to 1292 community members to assess need, perceptions, and opportunities for housing improvement for malaria control. Direct observations of 802 houses and surrounding environments were done to identify the actual needs and opportunities, and to validate the survey findings. A market survey was done to assess availability and cost of resources and services necessary for mosquito-proofing homes. Focus group discussions were conducted with key stakeholders to explore insights on the potential and challenges of housing improvement as a malaria intervention.

**Results:**

Compared to other methods for malaria control, housing improvement was among the best understood and most preferred by community members. Of the 735 survey respondents who needed housing improvements, a majority needed window screening (91.1%), repairs of holes in walls (79.4%), door covers (41.6%), closing of eave spaces (31.2%) and better roofs (19.0%). Community members invested significant efforts to improve their own homes against malaria and other dangers, but these efforts were often slow and delayed due to high costs and limited household incomes. Study participants suggested several mechanisms of support to improve their homes, including government loans and subsidies.

**Conclusion:**

Addressing the need for housing improvement is a critical component of malaria control efforts in southern Tanzania. In this study, a majority of the community members surveyed needed modest modifications and had plans to work on those modifications. Without additional support, their efforts were however generally slow; households would take years to sufficiently mosquito-proof their houses. It is, therefore, crucial to bring together the key players across sectors to reduce barriers in malaria-proofing housing in endemic settings. These may include government subsidies or partnerships with businesses to make housing improvement more accessible and affordable to residents.

## Background

Malaria is often recognized as a disease of poverty [1, 2]. At a global level, more than 90% of malaria cases and deaths are concentrated in the world’s poorest countries [3]. At more local levels, malaria is mostly concentrated in rural and poorer regions [4, 5], where poor housing is a common factor. Despite the changing behaviour of malaria transmitting mosquitoes that include early evening and outdoor biting [6, 7], still more than 80% of malaria transmission in sub-Saharan Africa occurs indoors [8], making house quality one of the key factors associated with malaria risk. Housing improvement such as screening windows and doors is one of the oldest reported malaria control interventions in the world, dating back to the nineteenth and twentieth century in Italy, Europe and the Americas [9, 10], and is linked to malaria elimination in those contexts [9, 11]. However, interest in housing improvement for malaria control declined following the discovery of insecticidal methods for killing mosquitoes, which were considered simpler, more affordable, and highly effective [9, 12, 13]. The intervention however started regaining interest following the emergence and spread of insecticide resistance in key malaria vectors; interventions not relying on insecticides were considered as one of the strategies to manage insecticide resistance [14].

Recent studies across sub-Saharan Africa have associated modest improvement in housing quality with decreased mosquito density and malaria incidence [15–18]. It has been noted, for example, that children living in improved houses made with brick walls, metal roofs, and closed eave space had 9% to 14% lower odds of being infected with malaria compared to those living in unimproved houses made with mud walls and thatched roofs across sub-Saharan Africa [15, 16, 18]. Other studies have also indicated higher densities of malaria vectors in unimproved houses compared to improved houses [4, 19–22].

Another line of research has been to assess whether community members living in malaria endemic settings understand the associations between housing structure and malaria transmission. In rural Tanzania, Kaindoa et al. [23] found that, while community members living in malaria endemic settings were aware of the risk of living in poorly constructed houses on malaria transmission, low-income levels and competing household priorities prevented them from improving their houses. A different study by Ogoma et al. [24], in urban Tanzania, found that a majority of community members associated housing improvement with lower risk of malaria transmission. On the contrary, a survey done in western Kenya to assess community knowledge and perceptions on malaria prevention and house screening reported low awareness of the impact of housing screening for malaria control [25].

Although previous studies suggest housing improvements can reduce malaria risk, there has been little effort in national and international malaria strategies to prioritize housing improvement. This is in part due to the poor understanding among stakeholders, of the need, magnitude and opportunities for housing improvement as a malaria control tool, and the perceived high costs of the intervention [26]. This current study was therefore aimed at understanding the perspectives of community members and other stakeholder groups regarding the need, magnitude and opportunities for housing improvement for malaria control and elimination in Tanzania.

## Methods

### Study site

**Fig. 1:**
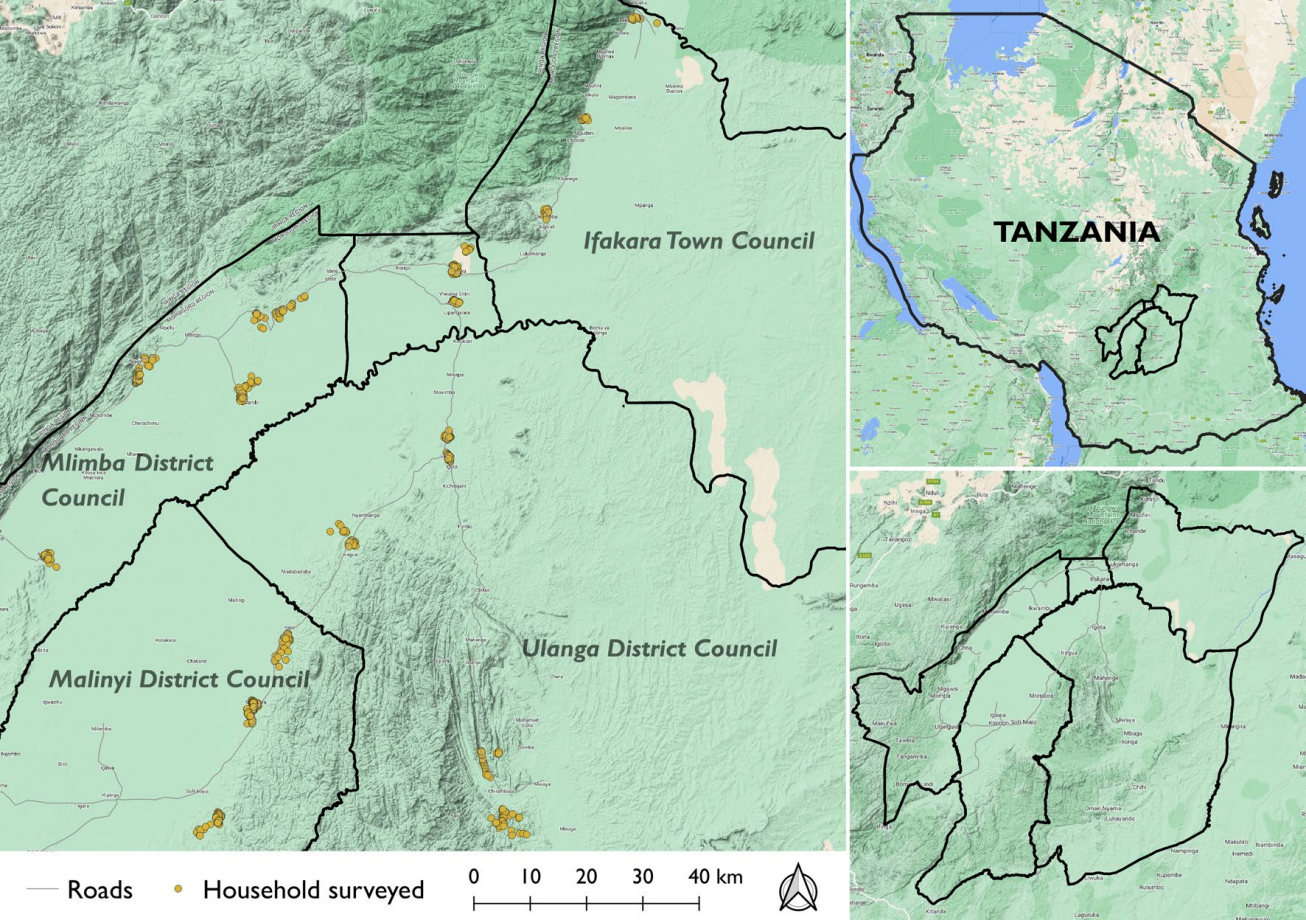
Map of study areas in the Kilombero valley, Tanzania

The study was conducted in 19 villages within the Mlimba, Malinyi and Ulanga district councils and the Ifakara town council, all in the Kilombero valley, southern Tanzania (Fig. 1). In Mlimba district, this study was done in Merera, Mofu, Njage and Namwawala villages. In Malinyi district Itete, Kalengakelo, Mtimbira and Sofi mission villages participated in this study. In Ulanga district Igumbiro, Iragua mission, Lupiro, Ebuyu and Mzelezi villages participated, and in Ifakara Town council Mlabani, Kibaoni, Ifakara mjini, Sululu, Mang’ula B, and Mkamba villages participated in this study. The councils have a diversity of settlements including urban, peri-urban and rural. A vast majority of the residents in rural and peri-urban settings are primarily farmers, but some also supplement that with other activities such as small businesses, fishing and livestock keeping. In the urban settings, many residents do various forms of entrepreneurship, supplementing it with farming. A majority of the houses in the area are made of brick walls and metal roof, and only a few have mud walls and thatched roofs [4, 23, 27].

### Study design and procedure

This study adapted a mixed method design [28, 29] to explore and assess preferences, need, perceptions and opportunities for housing improvement as a malaria control intervention in southern Tanzania. Quantitative component included two cross-sectional surveys, direct observations of houses and surrounding environments and market analysis for availability and cost of building materials. The exploratory qualitative component included focus group discussions (FGDs) with community leaders to explore their insights on the potential and challenges of housing improvement as malaria control interventions.

### Survey

Two rounds of surveys were conducted; the first round was done between November and December 2019, reaching 490 community members in ten villages. Details of the sample size selection are provided by Finda et al. [30]. This survey assessed community members’ awareness, knowledge, and preferences for alternative strategies to supplement current interventions for malaria control. The community members were provided with a list of six alternative strategies for malaria control and elimination including (a) larval source management (LSM), (b) spatial repellents (SR), (c) targeted spraying of mosquito swarms (SMS), (d) mass drug administration with ivermectin (MDA-IVM) to reduce vector densities, (e) release of modified mosquitoes (MM), and (f) housing improvement (HI).

The second round of survey was administered between March and June 2022 to 802 community members in 19 villages. This survey aimed to assess community perceptions, awareness, and available opportunities for housing improvement as a malaria control intervention. The villages in the second survey included the 10 villages in the first survey, but individuals surveyed were not necessarily the same. In both surveys, the households were randomly selected with guidance from household lists from the Ifakara Health and Demographic Surveillance System [31] and community leaders from the respective villages. Lists of households were identified in each sub-village, and a simple random formula was generated in excel. In case a household on the list no longer existed, the closest neighbour-household was visited and recruited to participate in the study. The surveys were administered to one adult household representative only after they had given written consent to participate. The survey was administered using Kobotoolbox™ software [32] on electronic tablets.

### Direct observations

Direct observations were done in each of the 802 households in the second survey to assess various houses and surrounding environmental conditions. Information on house characteristics included conditions of walls, roofs, floors, windows, and doors. Information about the surrounding environment was recorded up to 10-m radius surrounding the candidate house, and included presence of toilets, sources of domestic water sources, trash and potential breeding habitats for mosquitoes. The observation guide was incorporated in the survey that was administered to the household representatives and was done by a researcher conducting the main survey.

### Market survey

A market survey was conducted between September and November 2022 in one town in each of the four councils to investigate availability of building materials and services to respond to the identified needs for housing improvement. Altogether, 37 stores were identified, unevenly distributed between the four councils (Table 1). The stores were visited in-person where possible, and in other cases, phone numbers of store owners were obtained, and interviews were conducted through phone. The store owners were asked to provide general information about building products they sold, such as different product brands, their prices and popularity, and general information about their customers, such as where they come from and purchasing behaviours. Only stores that specialize in selling building materials, such as wire mesh, insect screens, cement, metal sheets, ceiling boards, woods and nails were surveyed. In addition to the assessment of hardware stores, in-depth discussions were also conducted with various vendors including store owners, carpenters, ironsmiths as well as masons to investigate cost for various house improvements services, varying from minor improvements such as window screening to major changes such as whole house constructions. Cost of the materials and services was determined.

**Table 1:** Availability of vendors of building materials in the Kilombero valley

Council	Town	Number hardware stores
Ifakara Town Council	Ifakara	25
Mlimba District Council	Chita	5
Malinyi District Council	Mtimbira	4
Ulanga District Council	Lupiro	3

### Focus group discussions

Eight FGDs were conducted with key stakeholders to discuss their insights on the potential of housing improvement as a malaria control intervention. Potential of housing improvement was discussed relative to other alternative tools for malaria control and elimination as previously described by Finda et al. [26]. The FGDs were done between December 2018 and December 2019. The key stakeholders were recruited from four groups that are all directly or indirectly involved with malaria control in Tanzania. They included policy makers, regulators, research scientists from two leading research institutions in the country, and community leaders from the villages where the surveys were conducted. A detailed description of these stakeholders is provided by Finda et al. [26]. A total of eight FGD sessions were held; two per stakeholder group, each including between six and ten participants. For the community leaders, men and women were separated to maximize participation by women [33]; but this separation was deemed unnecessary among the other stakeholder groups. A semi-structured discussion guide was used to facilitate the discussions. The sessions were audio-recorded and detailed notes were taken.

Field work was conducted by the first author (RMB), MFF, and trained research assistants; RMB and MFF facilitated the surveys, observations, FGDs and markets surveys, and the research assistants conducted the surveys and direct observations. The research assistants were provided with a 2-day training to familiarize them with the objectives of the study as well as the data collection tools. The structured surveys and direct observations were done in Swahili, and FGDs were done in both Swahili and English depending on the stakeholder group.

### Data processing and analysis

Quantitative data was analyzed using R statistical software version 4.2.1 [34]. Descriptive analysis was used to assess socio-demographic characteristics of the survey respondents, and summarize the characteristics of the houses, needed improvement and awareness of housing improvement as a malaria control intervention, and presence and cost of building materials and services. Binary logistic regression was used to examine the associations between the independent variables (wall type, roof type, window covers, door covers, social economic status, and location) and outcome variables (need and plan for improvement); odds ratio was calculated at 95% confidence intervals (CIs). The cost of house improvement needs per house was computed based on the market price of building materials, workmanship charges and local constructors’ experiences using bill of quantities (BoQ) for improving or building a standard house with an average of three sleeping rooms, four windows, and two doors. All cost were provided in TZS and converted into USD.

For the qualitative data, audio recordings from the FGDs were transcribed immediately following the discussions and translated from Swahili to English. The written transcripts were reviewed and analyzed using NVIVO 12 Plus software [35]. Objectives of the study and discussion guides were used to develop deductive codes, and inductive codes were generated through thorough reviews of the transcripts. Similar codes were grouped, and emergent patterns used to identify themes and concepts. Weaving approach [28] was used to present both quantitative and qualitative findings together. Perceptions of community members about housing improvement from the questionnaire were integrated with perceptions and the opinions of community leaders on the potential of housing improvement as a malaria control intervention. Where relevant, direct quotations from participants were used to support the claims.

## Results

### Socio-demographic characteristics of the study participants

A total of 1352 people participated in this study, including 490 community members in the first round of community-based survey, 802 in the second round of survey and 60 people participated in the FGDs. A detailed description of the community members who participated in the first round of survey and the FGDs is provided by Finda et al. [26, 30] and Mapua et al. [36]. As for the 802 who participated in the second survey, about two-third (60.6%, n = 486) were women, and 39.4%, (n = 316) were men. The average age was 45 years, ranging from 18 to 89 years. A majority (72.2%, n = 579) of the respondents had completed primary education (7 years of formal education), 13.1%, (n = 105) had completed secondary education and above (> 11 years of formal education), and 14.7%, (n = 118) had not received any formal education. Most (91.3%, n = 732) of the survey respondents were primarily small-scale farmers, but some also reported conducting small businesses, fishing, and animal husbandry on the side. The average reported monthly household income was 222,300.0 Tanzanian shillings (TZS), equivalent to $ 95.34 (In this case, $1 was converted to TZS 2332.43), and nearly a half (49.1%, n = 394) of the households were identified to live at or below national poverty line ($ 93.5) [37]. The average household size was 4.3 people, ranging from 1 to 27 people per household.

**Fig. 2:**
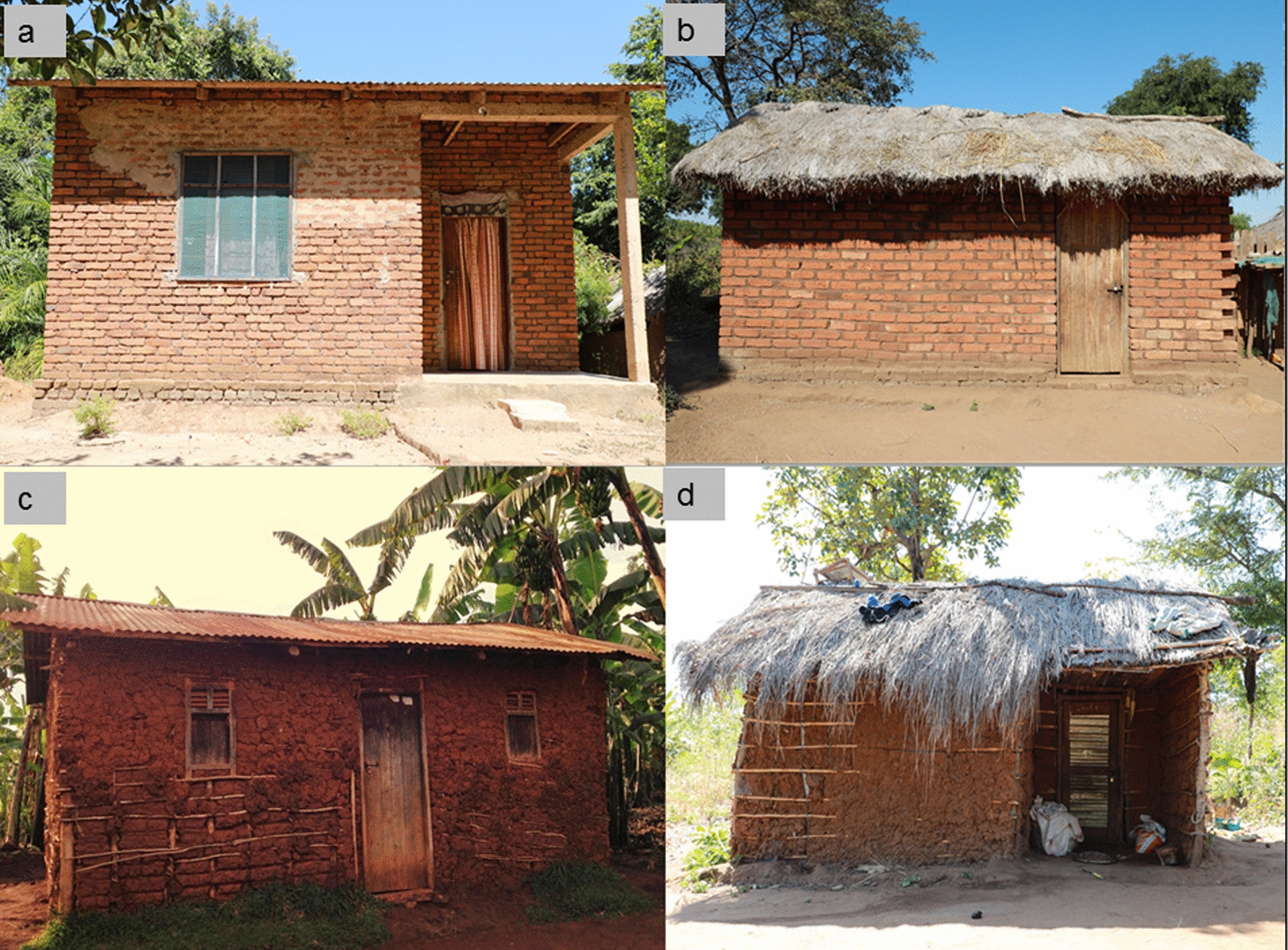
Common house types in the study sites: **a** brick walls with metal roof, **b** brick walls with thatched roof, **c** mud walls with metal roof, **d** mud walls with thatched roof

Generally, houses with brick walls and metal roofs were the most common type, comprising more than three-quarters of all surveyed houses (Fig. 2). Interestingly, most of these houses were found in urban areas (93.5%, n = 188) compared to rural areas (67.0%, n = 260). About half (50.1%, n = 402) of the households had flush toilets located outside of the main living area. Solar lamps were the main source of light in 40.1% (n = 322) of the households. Nearly a third (30.5%, n = 245) of the respondents used pump water from community centers, and about two thirds (69.3%, n = 556) used firewood for cooking.

### Common house characteristics

The surveyed houses had an average of 3 rooms, 4 windows, and 2 doors. Majority had brick walls (83.9%, n = 673), metal roof (80.7%, n = 647), and 95.8% (n = 768) of houses had windows. Common window covers included wire mesh (50.9%, n = 391), insect screen (45.6%, n = 350), and bricks (39.3%, n = 302), and common door covers were wood (76.2%, n = 611). While holes were observed in (74.1%, n = 569) of the windows, 60.3% (n = 484) of the doors and 51.9% (n = 416%) of the houses had open eaves with an average width size of 15 cm, ranging from 2 to 60 cm (Table 2).

**Table 2:** Characteristic of the surveyed houses

Variables	Category	All n (%)	Urban n (%)	Peri-urban n (%)	Rural n (%)
All houses		802 (100%)	201 (25.1%)	213 (26.6%)	388 (48.4%)
Major house type	Bricks wall and metal roof	626 (78.1%)	188 (93.5%)	178 (83.6%)	260 (67.0%)
	Mud wall and thatched roof	108 (13.5%)	6 (3.0%)	14 (6.6%)	88 (22.7%)
	Bricks wall and thatched roof	47 (5.9%)	2 (1.0%)	15 (7.0%)	30 (7.7%)
	Mud wall and metal roof	21 (2.6%)	5 (2.5%)	6 (2.8%)	10 (2.6%)
Wall type	Plastered bricks	246 (30.7%)	105 (52.2%)	67 (31.5%)	74 (19.1%)
	Unplastered bricks	427 (53.2%)	85 (42.3%)	126 (59.2%)	216 (55.7%)
	Mud	129 (16.1%)	11 (5.5%)	20 (9.4%)	98 (25.3%)
Condition of walls	No holes	474 (59.1%)	125 (62.2%)	133 (62.4%)	216 (55.7%)
	Holes	328 (40.9%)	76 (37.8%)	80 (37.6%)	172 (44.3%)
Roof type	Metal sheet	647 (80.7%)	193 (96.0%)	184 (86.4%)	270 (69.6%)
	Thatched	155 (19.3%)	8 (4.0%)	29 (13.6%)	118 (30.4%)
Condition of the roof	No holes	579 (72.2%)	123 (61.2%)	171 (80.3%)	285 (73.5%)
	Holes	223 (27.8%)	78 (38.8%)	42 (19.7%)	103 (26.5%)
Windows cover*	Wire mesh	391 (50.9%)	134 (66.7%)	118 (57.6%)	139 (38.4%)
	Insect screens	350 (45.6%)	117 (58.2%)	103 (50.2%)	130 (35.9%)
	Bricks	302 (39.3%)	60 (29.9%)	86 (42.0%)	156 (43.1%)
	Uncovered	85 (11.1%)	25 (12.4%)	22 (10.7%)	38 (10.5%)
	Curtains/clothes	80 (10.4%)	18 (9.0%)	21 (10.2%)	41 (11.3%)
	Wood/bamboo	75 (9.8%)	17 (8.5%)	18 (8.8%)	40 (11.0%)
	Others	36 (4.7%)	18 (9.0%)	9 (4.4%)	9 (2.5%)
Condition of the Windows cover	No holes	199 (25.9%)	70 (34.8%)	57 (27.8%)	72 (19.9%)
	Holes	569 (74.1%)	131 (65.2%)	148 (72.2%)	290 (80.1%)
Entry door cover*	Wood/bamboo	611 (76.2%)	161 (80.1%)	154 (72.3%)	296 (76.3%)
	Metal sheet	194 (24.2%)	35 (17.4%)	58 (27.2%)	101 (26.0%)
	Grill	67 (8.4%)	38 (18.9%)	17 (8.0%)	12 (3.1%)
	Uncovered	35 (4.4%)	6 (3.0%)	11 (5.2%)	18 (4.6%)
	Bricks	16 (2.0%)	3 (1.5%)	5 (2.3%)	8 (2.1%)
Condition of the doors cover	No holes	318 (39.7%)	92 (45.8%)	78 (36.6%)	148 (38.1%)
	Holes	484 (60.3%)	109 (54.2%)	135 (63.4%)	240 (61.9%)
Eaves space	Open eaves	416 (51.9%)	83 (41.3%)	103 (48.6%)	230 (59.3%)
	Closed eaves	385 (48.1%)	118 (58.7%)	109 (51.4%)	158 (40.7%)
	Average open eave width (range)	15 (2–60) cm	8 (2–40) cm	14 (3–60) cm	15 (2–60) cm
Ceiling	Not present	727 (90.6%)	164 (81.6%)	200 (93.9%)	363 (93.6%)
	Present	75 (9.4%)	37 (18.4%)	13 (6.1%)	25 (6.4%)
Ceiling material	Gypsum	38 (50.7%)	24 (64.9%)	11 (84.6%)	3 (12.0%)
	Wood	26 (34.7%)	10 (27.0%)	0 (0.0%)	16 (64.0%)
	Nylon	11 (14.6%)	3 (8.1%)	2 (15.4%)	6 (24.0%)
Condition of ceiling	No holes	59 (78.7%)	32 (86.5%)	13 (100%)	14 (56.0%)
	Holes	16 (21.3%)	5 (13.5%)	0 (0.0%)	11 (44.0%)
Floor-type	Mud	449 (56.0%)	60 (29.9%)	115 (54.0%)	274 (70.6%)
	Cement	324 (40.4%)	125 (62.2%)	89 (41.8%)	110 (28.4%)
	Tiled	29 (3.6%)	16 (8.0%)	9 (4.2%)	4 (1.0%)

*Percentages add to more than 100% because of multiple selections

### Definition of a mosquito-proof house

Community leaders associated ‘modern’ houses with being mosquito-proof. When asked to define what a mosquito-proof house meant to them, the leaders termed it as a modern house (*nyumba ya kisasa)*, and listed many features including large house size, large windows, screened doors and windows, brick walls, metal roof and electricity. The leaders explained that well ventilated lighted and uncluttered indoor environment would be unsuitable for mosquitoes, as expressed by these two leaders:

*Three main important things are brick walls and metal roofs and big windows. Those are the basic, other things can be added with time. You also need to put netting on the doors and windows, and then another big addition is also to put electricity. Mosquitoes do not like electricity. Then if you have electricity, you can also have a fan, and a fan chases mosquitoes away, they do not like a fan. I tell you, if a house is well lit with big windows, mosquitoes can never have a chance.* (Male community leader)

*For me, a modern house is a brick house that has big enough windows that can allow air and light in. It has enough space to sit and cook. It has a bathroom and a sitting room. It is a house that people can feel comfortable to stay in and cook, eat, and relax. That is what I think is a modern house.* (Female community leader)

When asked whether or not their current houses provided protection against malaria vectors, a majority (88.4%, n = 709) of the survey respondents said no, and only (11.6%, n = 93) believed that their houses provided protection. Of those that said their houses did not provide protection, they described their houses as having a lot of holes in the walls and roofs through which mosquitoes get inside. The houses were also dark and cluttered hence providing a lot of hiding places for mosquitoes. One community leader said:

*I tell you that these traditional houses have a lot of hiding places for mosquitoes. Also, you see people normally put very small windows, or they do not put any windows at all, or sometimes they have small windows, but they completely cover them with clothes or bricks, as a result, it is always dark inside, and we all know that mosquitoes like the dark.* (Male community leader)

The community leaders further explained that their houses are generally very small, forcing people to conduct household chores outdoors, exposing them to the risk of outdoor malaria transmission. It was in some cases difficult to use currently available mosquito control interventions such as bed nets or insecticide-sprays due to the small size and structures of the houses, or the holes in the houses through which mosquitoes can enter freely. One community leader explained the difficulty using insecticide spraying as follows:

*“It is quite difficult to kill mosquitoes in these houses as however many times you spray the insecticides, mosquitoes keep coming back because these houses have a lot of holes, so new mosquitoes can keep coming in.”* (Male community leader)*.*

### Perceptions of housing improvement for mosquito control

**Fig. 3:**
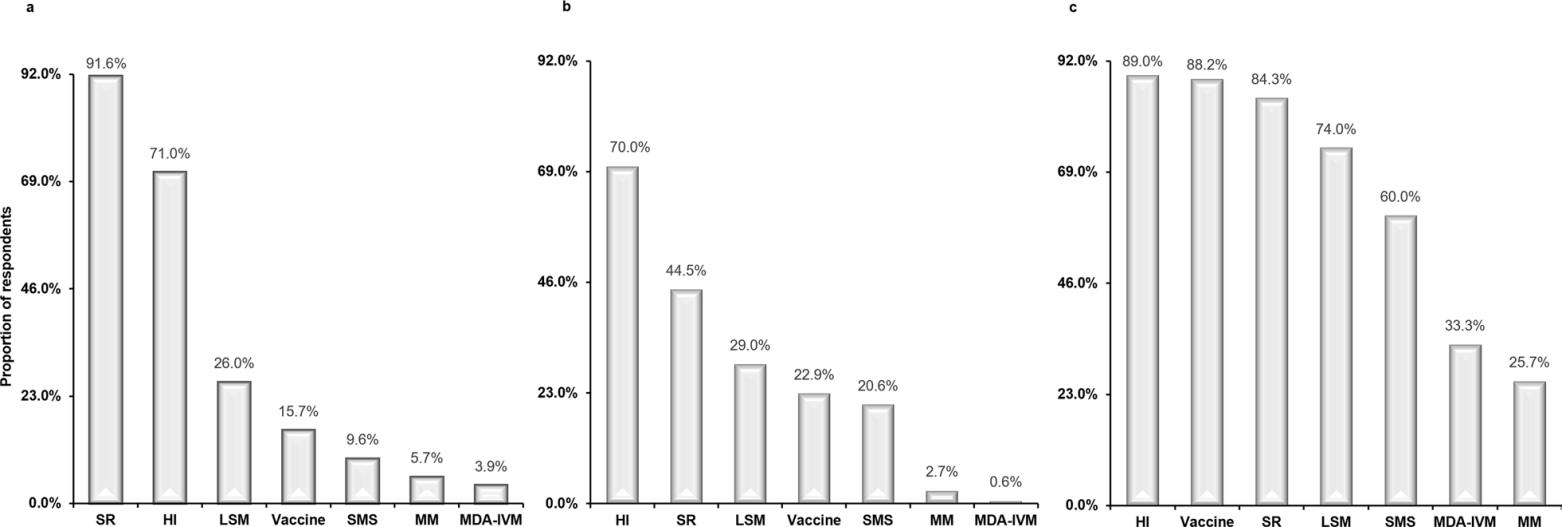
Awareness, knowledge and preference of alternative tools for malaria control and elimination among community members in southern Tanzania: **a** Awareness of housing improvement for mosquito control, **b** knowledge of how housing improvement works in mosquito control, **c** preference for housing improvement for malaria control

When presented with several alternative strategies for mosquito control, a majority (91.6%, n = 449) of the community members that participated in the first survey reported awareness of the potential of housing improvement in controlling malaria vectors. Additionally, 70.0% (n = 343) of the community members had correct knowledge of how housing improvements works in malaria control, and 89.0% (n = 436) preferred housing improvement compared to the other alternative tools (Fig. 3). Preference for housing improvement was also widely expressed during the FGDs with the key stakeholders, where most of the community leaders discussed that all other strategies would not be fully effective in controlling or eliminating malaria if people continue to live in poor houses that do not offer any protection against mosquitoes. The leaders further explained that the potential of housing improvement made the most sense to them compared to the other strategies, as it provides protection against not only mosquitoes but also other diseases and dangers. Two leaders elaborate these concerns here:

*For me to live well and feel safe I need to be in a nice house, made with bricks and metal roof, with big space and big windows with net. I like that it will protect me from not just mosquitoes, but also many other diseases and other dangers like snakes and flooding.* (Male community leader)

*I like improving or building houses for people so that they are safe from mosquitoes. All these other solutions are really good, but if people do not have houses that protect them then I do not think that anything will work 100%. So, I would advise that we put people in protective houses and then add other solutions.* (Female community leader)

The high preference for housing improvement for malaria control, however, was not reflected among other stakeholder groups, who feared that it would not be affordable or sustainable for a low-income country like Tanzania. Additionally, housing improvement was seen as insufficient in effectively controlling malaria as policy makers believed that the risk of malaria transmission was not confined inside houses as this policy maker explained:

*I do not at all agree with the technology of improving houses for people because I do not think that malaria is only transmitted in the house; people can get malaria anywhere mosquitoes are, so I do not see the point of focusing on just houses. I think we should focus on getting rid of mosquitoes, not just keeping them outside the house.* (Male Policy maker)

There were stakeholders that proposed focusing on environmental improvements rather than just the house noting that malaria transmission will still persist if the surrounding environment is mosquito-friendly as this regulator said:

*It does not matter how nice your house is, if the environment is suitable for mosquito reproduction, they will always be there. I would advise to focus on improving the environment and the sewage system, to destroy all the places mosquitoes can breed or hide.* (Male regulator)

Furthermore, were participants, particularly scientists who explained that the potential of housing improvement for malaria control has not thoroughly investigated, hence inadequate evidence for it. These stakeholders discussed that it would not be advisable for the government to be directly involved in housing improvement for malaria control as this scientist said:

*I do not think that this is an intervention that the government can invest in directly, maybe more indirectly. House improvement is a part of development, it happens naturally… But the problem is that we have not been documenting the impact of these changes in terms of malaria control, so we cannot really say for sure how this has contributed in malaria control.* (Male scientist)

In the second community-based survey however, 69.6% (n = 558) were aware that improved housing protects against malaria. When asked about their source of this information, a majority of the respondents said that they knew from their daily experiences, but others listed family and relatives as well as hearing about it in television and radio. For those that disagreed that improved housing provides protection against malaria, the main reasons given were that it was that improved houses alone would not provide complete protection against malaria, as mosquitoes could still get in through open doors or windows, and due to tradition, people would still spend time outdoors.

### Housing improvement needs among community members

Most (91.6%, n = 735) of the surveyed community members expressed the need for some improvement to make their houses mosquito-proof. Most of the improvements needed were on adding or repairing window screens (67.2%, n = 494), repairing walls (43.0%, n = 316), adding or repairing doors (36.7%, n = 270), and changing or repairing roof (32.2%, n = 237). Only 17.0% (n = 125) of the surveyed respondents needed their whole houses reconstructed to provide any protection from mosquitoes (Table 3). When asked whether they had plans to make the needed improvements, 87.6% (n = 644) reported planning to do so in a period of between 1 and 5 years. Nearly three quarters (73.3%, n = 588) of the community members listed affordability as the main reason for the delays in making the needed house improvements.

**Table 3:** Community-reported housing improvement needs

Improvement needs	Category	n (%)
Windows (n = 494, 67.2%)	Adding or repairing screen	450 (91.1%)
	Adding wood or metal protection	57 (11.5%)
	Adding glass cover	40 (8.1%)
	Increasing windows size	32 (6.5%)
	Other window improvement	16 (3.2%)
Walls (n = 316, 43.0%)	Plastering or repairing walls	251 (79.4%)
	Closing eave space	99 (31.2%)
	Painting walls	41 (13.0%)
	Other wall improvement	5 (1.6%)
Door (n = 270, 36.7%)	Adding wood or metal cover	112 (41.6%)
	Adding or repairing screen	99 (36.7%)
	Adding wood or metal frame	49 (18.2%)
	Increasing door size	22 (8.2%)
	Other door improvement	18 (6.7%)
Roof (n = 237, 32.2%)	Adding ceiling	120 (50.6%)
	Repairing roof	90 (38.0%)
	Better roof	45 (19.0%)
	Other roof improvement	13 (5.5%)
Whole house (n = 125, 17.0%)	Reconstruct a whole house	109 (87.2%)
	Other house improvement	16 (12.8%)

The issue of affordability also dominated the FGDs with community leaders, who explained that everyone wishes to live in an improved house, but the cost is too high. Some of the costliest materials were said to be doors, windows, and metal roofs. For example, one community leader elaborated that when people build modern houses, they normally put a lot of big windows and multiple doors to ventilate their houses. But since windows are expensive, people often temporarily cover the window openings with bricks until they can afford to install proper windows or doors as this community leader elaborated:

*“If people cannot afford to screen their windows, then they normally cover them with bricks. You know our biggest challenge is poverty. I know people like to live in nice houses with big windows that can allow ventilation, we like that very much. But if you have very little money, then you just have to deal with what you have, and that is why you see a lot of doors and windows that are not screened. We know that screening would provide protection against mosquitoes, we just cannot afford it.”* (Male community leader)

### Availability and cost of building materials

Altogether a total of 37 stores were identified and contacted in four towns in the four councils in the Kilombero valley in southern Tanzania. Ifakara town, the most urban of the four councils, had the highest number of stores. Many of the Ifakara town stores sold both wholesale and retail, and their customers came from across the Kilombero valley, including store owners in the more rural councils. The stores in the more rural towns were smaller in size and sold just retail. Their customers were reported to be from within their surrounding communities.

**Fig. 4:**
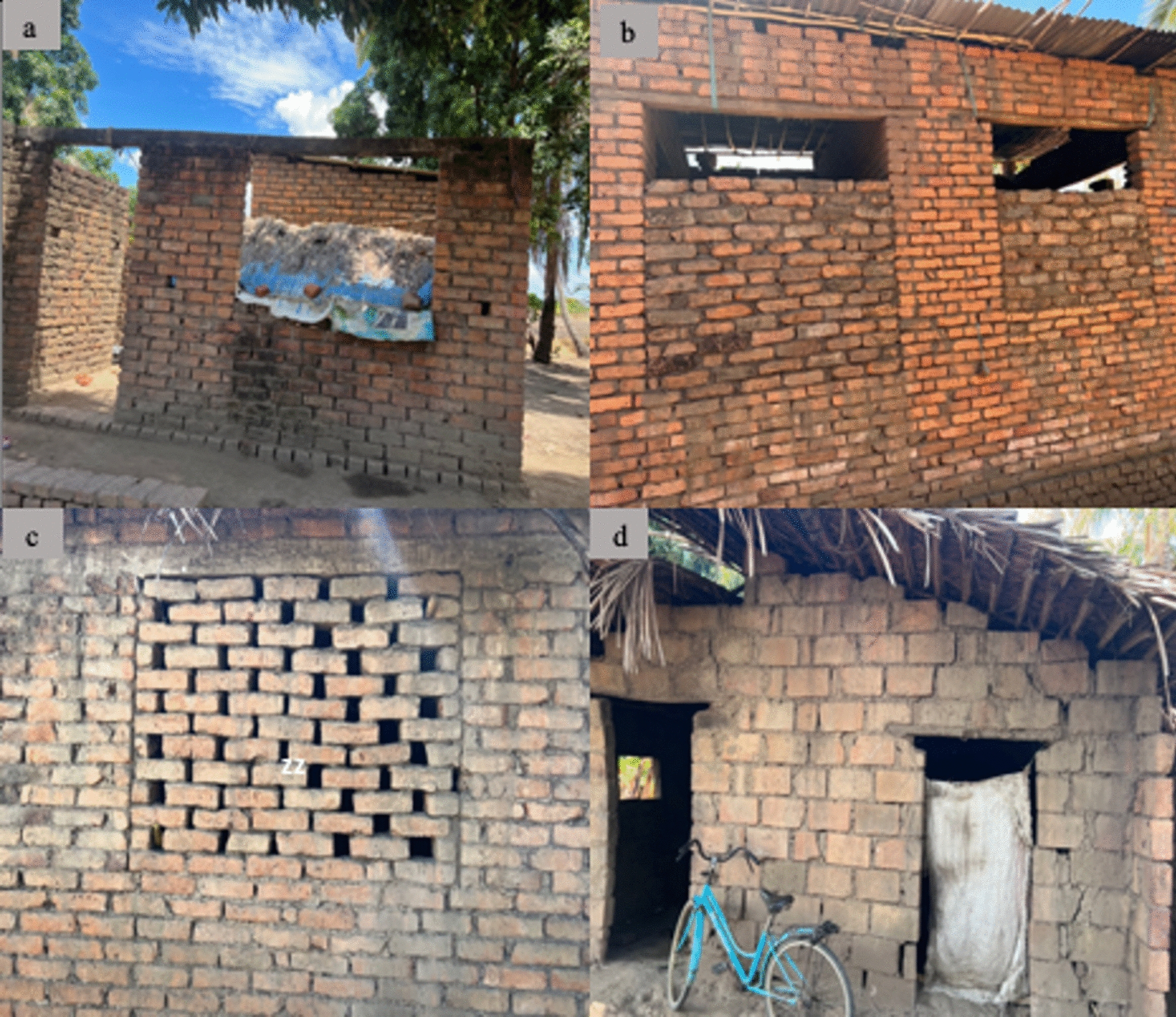
Examples of improved but incomplete houses that people were residing in: **a** un-roofed house, **b** bricks on windows with small gaps on top, **c** holes in the walls and **d** no door covers, open eaves

The store owners explained that their highest selling season for building materials was immediately following the harvesting season. Most popular products were cement, metal sheet for roofing, and insect-screens for windows and doors. The interviews revealed that after selling their farm products, people would often start building bigger houses, but they would often not be able to complete these within the season, and would either complete parts of the houses or cover windows and doors with bricks and defer to the following harvesting season (Fig. 4). The store owners also reported that people would often purchase building materials at irregular intervals, depending on when they get funds. Due to this, building a single house could take years to complete. However, it was common for people to move into unfinished house and keep on completing as they live in it (Fig. 4).

Like the store owners, builders and carpenters also explained that often people build their houses in steps, depending on when they can afford. They indicated that most people built the walls with bricks and mud, and later on plaster with cement and sand. This was said to be more affordable than building with bricks and cement. The builders said that the most expensive materials for people were cement, metal sheets, and door and window frames and covers. Bricks were said to be locally manufactured and affordable. They further indicated that sometimes it could take them years to complete a house construction depending on when the owners were able to secure building materials or afford to pay for the building services.

Estimated price for conducting various house improvements is provided on Table 4. This price is calculated for an average house surveyed, which has an average of three rooms, four windows, two outlet doors, and 4 household members. The price presented is a combination of the price of materials in the stores as well as the cost of providing the services obtaining from discussions with the builders. Increase in complexity of improvement needs was directly associated with increase in cost for the planned. For example, minor improvements such as screening windows and doors, and closing eave gaps reported a modest cost ranging between $31.5–54.5. Medium improvements such as adding windows and door covers were estimated to cost between $166.9–463.3, and major improvements, in this case constructing a new house of the average size was estimated to cost between $4590.9–4967.0 (Table 4).

**Table 4:** Estimated cost for various house-improvements

Category	Estimated cost per average house (USD)
Adding or repairing window screens	31.5–34.4
Adding protective bars on windows	208.7–240.2
Adding window glass covers	394.7–463.3
Increasing window sizes	243.1–274.6
Plastering or repairing walls	351.8–386.1
Close eave gaps with bricks	42.2–47.8
Painting walls	540.1–557.7
Adding wood or metal door shutters?	166.9–171.6
Adding or repairing door-screens	42.9–54.5
Adding door frames	103.0–128.7
Increasing door sizes	244.6–269.9
Adding ceiling	310.2–315.7
Repairing roofs	480.9–750.4
Changing roofs	1491.2–1611.4
Constructing a whole house	4590.9–4967.0

### Factors associated with the need for housing improvement for malaria control

In a univariate analysis, the need for housing improvement was significantly associated with the type of walls, window covers, door covers, and location of the houses (Table 5). Households with unplastered brick walls were more than five times more likely to demand housing improvement than those with plastered walls, and houses with mud walls were nearly four times more likely to need improvements compared to those with plastered walls. In terms of windows, those that had been covered with bricks were more than six times more likely to need improvements compared to screened windows. Doors that were covered with grill only or metal sheets only were also significantly more likely to need improvements compared to the traditional wood covers. In terms of socio-economic status, survey respondents classified as poor were more than twice as likely to need housing improvement compared to those classified as less poor. Finally, houses in rural settings were also more than twice as likely to need improvements compared to those in rural settings (Table 5).

**Table 5:** Factors associated with the need for housing improvement

Variable	Category	Univariate	
		Odd ratio	P-value
Wall type	Plastered bricks	1	–
	Unplastered bricks	5.71 (3.15–10.35)	< 0.001
	Mud	3.84 (1.59–9.31)	0.003
Roof type	Metal sheet	1	–
	Thatched	1.84 (0.86–3.94)	0.116
Windows cover	Insect screen	1	–
	Wire mesh only	1.20 (0.77–1.87)	0.408
	Bricks only	6.22 (2.77–13.99)	< 0.001
	Wood only	0.65 (0.34–1.26)	0.202
	Curtains only	2.43 (0.85–6.99)	0.099
	Uncovered	1.60 (0.70–3.69)	0.266
Door cover	Wood	1	
	Grill only	0.26 (0.15–0.47)	< 0.001
	Metal sheet only	5.27 (1.89–14.68)	0.001
	Uncovered	1.00 (0.23–4.41)	0.998
Social Economic Status	Less poor	1	–
	Poor	2.58 (1.44–4.63)	0.001
	Poorest	2.20 (1.09–4.43)	0.027
Location	Urban	1	–
	Peri-urban	0.80 (0.44–1.47)	0.480
	Rural	2.27 (1.19–4.32)	0.013

### Options for additional support

Community leaders explained that people in the community were making great efforts in improving their homes, however, if left for people to do this on their own, the poorest in the communities would not afford to improve their houses fast enough to keep up with the government’s efforts to eliminate malaria. The community leaders discussed various options that the government could consider helping its citizens. One of the popular options was for the government to provide people with loans to build or improve houses. The participants elaborated that the government could work with community leaders to help identify the poorest people in the community and provide them with loans to build or improve houses, and people would slowly pay back; as this participant said:

*“I would advise the government to give house loans, especially to the very poor people so that they too can have houses that they can stay in and not be forced to spend half of the night outside. In the villages, most people are very poor and such help would be really good for them.”* (Female community leader)

There were participants who argued however, that it would not be easy for the government to single out the poorest people and help just those; these participants suggested that the government reduces the cost of building materials so that more people could afford to build better houses or improve their houses, explaining that if the building price is subsidized, then everyone could afford to improve their homes. The leaders took examples from various programs that the government has done to help its citizens achieve better homes. One example was Tanzania’s Rural Energy Agency (REA) [38], whose aim is to facilitate availability and access to affordable electricity in rural settings in Tanzania. The leaders explained that if the government has been able to subsidize electricity costs so that the poorest in the country can afford it, the government could use similar approach and subsidize building costs as these participants said:

*“It would be good if the government could help. You know, like they are helping with REA electricity, they look at people that are poor and they reduce the cost of installing electricity, so that everyone can afford. In the past only rich people could afford electricity, but now they have made it easy for us, so now we all have electricity. I think they can definitely do this with housing too. I am not saying that they should give us everything, but they should help make it easy for everyone to build a modern house.”* (Male community leader)

*“I think it would be very difficult for the government to help one person at a time. I think it would be easier for the government to just subsidize the costs of building materials, then everyone can afford to build. It is better than giving loans to individual people, which you don’t even know that they will use them for building. Some people can use the money to buy food or send their kids to school, will you blame them?”* (Male community leader)

Other participants suggested that the government should rather build standard houses and rent them to people at affordable prices or giving people an opportunity to refund. The participants gave an example of “*Nyumba ni Choo”* (*A house is only as good as the toilet is*), a country-wide campaign to improve health status of the people by controlling water, sanitation and hygiene related diseases [39]; the government in collaboration with international partners had built proper latrines for the poorest people in the communities, and people paid back slowly. Similar approach was proposed for housing improvement, in which the community leaders proposed the government to identify the neediest in the communities and assist them in improving their houses, and then the community members would pay back slowly. One community leader explained this process below:

*I know there was a time, a few years back when people came and gave us loans to build modern toilets. They built the toilets for the people; they brought their own builders and the materials, and then they asked people to pay them back slowly. Now most people in the villages have modern toilets but very poor houses... The government can maybe build the houses, and people can repay the government slowly, everyone can pay according to what they can afford.* (Female Community leader)

## Discussion

This is the first study that has undertaken a thorough assessment of the magnitude, types of housing improvement needed for malaria control, and locally available and acceptable opportunities to respond to the need. This study indicates a majority of the surveyed households need relatively modest improvement to make their houses malaria proof. The most popular needs included adding window screens, installing better windows and doors, and covering holes on walls and roofs. Such improvements have been shown to vastly reduce the risk of malaria transmission in Tanzania [23], Equatorial Guinea [16], Gambia [19, 40], and Uganda [18], among other countries. Lower odds of malaria infection and fewer malaria cases have also been reported in people who live in improved houses [15, 41]. Of all the houses surveyed, only 17% needed to be reconstructed to be malaria-proof. This is a crucial finding, as this need for full-house construction is much lower than had been anticipated by policy makers, regulators and scientists. Additionally, the cost for reconstructing a full standard-size house was also estimated to be less than $5000, which is also relatively low cost, considering the potential benefit an improve house has, which spans far beyond malaria control [42–44]. Additional cost-effectiveness studies are needed to demonstrate the overall health benefits of people living in improved houses.

The definition of an improved house or modern house was uniform among the surveyed community members; it included houses that were built with brick walls, metal roofs, screened doors and windows, and closed eaves. Electricity was also listed as an essential. It was also evident in this study that community members are making incredible efforts to modify their houses to fit this ideal of an improved house, as more than three-quarters of the houses were what Tusting et al. [41] referred to as modern houses, although a majority lived at or below the poverty line. Although this drive to improve housing condition has been observed across the country [45, 46], however, when left to just the community members alone, the improvements take a long time to complete due to financial reasons, as community members reported that on average it could take them up to 5 years to malaria-proof their houses. Additional support to these community members could help improve and speed up malaria control and elimination efforts. Lindsay et al. [47] proposes that a range of facilitators, both in the public and private sectors need to be involved when discussing the prospects of housing improvement. These may include microfinance institutions, government ministries, town planners, architects, public health inspectors, and community members among others, to ensure that citizens live in disease-free houses [47]. Together these key players can come up with housing improvement solutions that are both affordable and sustainable for both the country and the affected communities.

Community members were aware of the value of an improved house in reducing the risk of malaria transmission; they linked small and unlit houses to increased risk of exposure to malaria vectors as they provide a suitable environment for mosquitoes to hide, and forced people to spend most of their evening and early night hours outdoors, exposing them to malaria vectors. This awareness of risk of outdoor malaria transmission is supported by a study done in the same settings which indicated that the highest risk of exposure to malaria transmission occurred during the early night hours when a majority of people were outdoors in peri-domestic settings [5]. Despite the existing awareness of the value attached to improved housing, the major concerns for the delay on the housing improvement were associated with low and or highly cyclical income; people are only able to afford building during the harvesting season when they can sell their farm products.

Interestingly, a previous study by Kaindoa et al. [23] in the same villages also indicated low income as the main factor associated with delays in housing improvement.

Even in the cases where considerable investments in housing improvements were made, it was observed that houses with brick walls or metal roofs failed to provide full protection against malaria vectors since many had holes on the walls, doors, windows, and roofs. Many houses were also found unfinished, albeit people lived in them due to high construction costs. For example, lack of proper window and door covers forced many households to build bricks to temporarily cover where windows and doors could have been in order to provide protection from other dangers, such as animals and burglars. Smaller holes were then intentionally left on walls to let light and air in, and these also serve as potential mosquito entry points. The fact that many people live in somewhat improved houses may give misguided hope that they are in a malaria-protective environment, but these houses may still expose to people to as much risk as if they lived in unimproved houses. In order to ensure rapid gains in malaria control and elimination efforts, it is crucial for governments and malaria control agencies to supplement the efforts that people make in malaria-endemic settings towards improving their houses.

Community members stressed that support from the government would be imperative in helping people to live in a safe and protective environment. They offered several recommendations for the government and other relevant agencies to help improve their houses more quickly. These included providing building loans, subsidizing the cost of building materials, or building standard houses and renting to the poor at an affordable price. However, policymakers were strongly opposed to the thought of the government assisting communities in improving their houses, claiming that it is neither affordable nor sustainable for the government, and that housing improvement alone would not be sufficient to eliminate malaria. However, this lack of support from the government officials is most likely due to lack of information on (i) the actual magnitude of the need for housing improvement in malaria-endemic settings in the country, (ii) the role that housing improvement has played in malaria elimination in other settings in the world [9, 11, 41], or (iii) the evidence of how various housing improvement strategies have resulted in reduction in risk and severity of malaria [41]. It is crucial to ensure that these decision makers at the government level are provided with adequate information on these aspects of housing improvement for malaria control.

In a previous study with the same stakeholders, it was noted that decision-makers at the national and community level rely upon information from scientists to make informed decisions related to malaria control [26, 30, 36]. It therefore lies on the shoulders of the scientists to generate and adequately disseminate information on the potential of housing improvement for malaria control and opportunities for helping communities in endemic settings speed up the efforts they are already making in malaria-proofing their houses.

## Limitations of the study

The major limitation of this study is that it was conducted in a community that is relatively homogeneous in southern Tanzania, therefore, these findings may not be generalizable to the whole country or other malaria endemic settings in Africa. Still, these findings offer a baseline from which further studies can be developed in other malaria endemic settings, to explore the need and potential of housing improvement to help speed up malaria control and elimination efforts. Another limitation of this study was that, while it has been increasingly reported that houses with brick walls are more protective compared to mud-walled houses, however, the actual risk that different house types or conditions pose was not assessed in this study. Moreover, community members described an ideal malaria-proof house to have enough space for people to be able to spend more time indoors. However, this study did not dive deep in defining what ‘enough space’ meant, neither did it measure the actual size of the houses observed.

## Conclusion

The study found that housing improvement was a well-understood and supported intervention for malaria control among the rural communities in southern Tanzania. The majority of survey respondents who needed house improvements cited the need for window screening, repair of holes in walls, door covers, closing of eaves, and better roofs. Community members were willing to invest in improving their homes but were limited by financial constraints. Whereas most households surveyed needed only modest modifications, the high poverty levels meant that without additional support, it may take years for these households to obtain malaria-proof their homes. The study participants suggested government loans and subsidies as potential mechanisms of support to improve their homes against malaria.

Also, due to inadequate evidence of the potential of housing improvement for malaria control, this strategy lacks support among the country’s top decision makers. It is, therefore, highly necessary for scientists to generate and disseminate knowledge and evidence on what housing modifications can result in optimal success in providing protection against malaria and other infectious diseases. Finally, it is important to bring together all the key players in the housing sector to reduce barriers to malaria proofing housing in an endemic setting.

## Data Availability

All data for this study will be available upon request.
